# A simulated patient study to evaluate community pharmacist assessment, management and advice giving to patients with asthma

**DOI:** 10.1186/s40545-020-00294-4

**Published:** 2021-01-12

**Authors:** Sanah Hasan, Lujain Al Oum, Nageeb AbdulGalil Hassan

**Affiliations:** 1 Department of Clinical Sciences, College of Pharmacy and Health Sciences, Ajman University, Ajman, United Arab Emirates; 2 Department of Medicinal Chemistry, College of Pharmacy, University of Sharjah, Sharjah, United Arab Emirates; 3 Department of Pharmaceutical Sciences, College of Pharmacy and Health Sciences, Ajman, United Arab Emirates

**Keywords:** Community pharmacist, Asthma, Advice, Simulated patient, Mystery shopper, United Arab Emirates

## Abstract

**Background:**

Research has shown that there is an increase in the global prevalence of asthma. Pharmacists are well positioned to screen and refer patients for better management of asthma. This study aimed to evaluate community pharmacists’ ability to assess the 3 C’s (*Control, Compliance, Complications*) and offer *Management* and *Advice* for patients with asthma in the UAE.

**Methods:**

Three fifth year pharmacy students role played a mystery shopper visiting community pharmacies and requesting symptom relief from uncontrolled asthma. Incidence of cough syrup and reliever inhaler supply, physician referral rate, correction of inhaler technique, and counseling on the medications, adherence to the medications and adverse drug reactions were calculated.

**Results:**

One hundred, ninety five pharmacies were visited, 27% of pharmacists asked about the need for cough syrup and 60% asked about the need for albuterol inhaler. Only 26% asked about other medications. Less than 20% assessed inhaler technique, only *one* pharmacist asked about regular use of preventer medications and if the patient was adhering to them and only 16% asked about side effects from medications. Most pharmacists (67%) supplied at least one of the medications, while 65% referred the patient to a physician. Only 21% gave information about correct inhaler technique, the majority (> 60%) being incomplete, only 16 pharmacists gave information about asthma and its triggers; the majority (63%) being incomplete. One third of the pharmacists counselled the patient on the medications with *one* giving complete information.

**Conclusions:**

The study highlighted suboptimal assessment of *control, compliance* to preventer medications and *complications* of asthma and the medications that treat it. It also highlighted suboptimal *Management* and *Advice giving* and counseling on medication use by pharmacists. Training pharmacists in all aspects of asthma handling is clearly indicated. Despite the high rate of correct patient referral to a physician noted in this study, there is risk to it, as patients might not actually go to see their physician and continue to depend on symptom relief for the management of their asthma. This study highlighted the importance of improving patient education and information seeking attitude.

## Background

Asthma is a chronic inflammatory condition affecting the airways of more than 300 million people worldwide [1]. It is associated with significant clinical, economic and humanistic burden on the individual and society. Several studies have shown that there is an increase in the global prevalence of asthma [2]. Therefore, it is vital that health professionals help patients better manage their condition [3]. Healthy People 2020 urges health practitioners to follow asthma guidelines, devise written asthma management plans, and provide proper patient education to improve disease outcomes [4]. Pharmacists are well positioned to screen patients with signs and symptoms of asthma, and to help diagnosed patients achieve better control and management of their condition [5–9].

### Theory of assessment

Herrier et al., suggested a general approach (The *chronic disease follow-up visit history*) of pharmacist interviewing patients for chronic disease follow-up [10]. It is structured around the “3 C’s” schemata for evaluating the quality of care in the patient with chronic disease. Here, for all chronic diseases, there are three areas to be evaluated. *Control* of the disease, *compliance* with the therapeutic regimen, and *complications* due to the disease and/or the drugs used in its treatment. In this model, there is a general open-ended question to assess each of the three areas of interest, followed by disease-specific open-ended questions for probing additional information. Furthermore, the pharmacist can periodically ask about a fourth C, *Concern*: “What kind of concerns do you have about your condition and/or its treatment [10].

### Asthma assessment

In the community setting, the pharmacist will first need to confirm a diagnosis of asthma before further intervention. After this confirmation, and according to the above approach [10], the pharmacist is expected to assess the three C’s of the condition: *Control* of asthma by asking about the degree of control of symptoms, frequency of reliever medication use, and other medications are being used. The pharmacist would also assess the degree of *Compliance* with regular preventer medications, and the appropriateness of inhaler use. Thirdly, the pharmacist would ask about *Complications* from the lack of control of the condition and from side effects of medications.

After patient assessment, pharmacists need to provide *Management* for the condition, such as dispensing medications, referring the patient to a physician for follow up, and giving advice to the patient. Rate of supply of symptom relievers, e.g., albuterol and/or cough syrup, referral rate to a physician, and the quantity and quality of the advice the pharmacist offers to the patient on asthma, its triggers, the medications used, inhaler technique, adherence and adverse drug reactions are assessed.

### Simulated patient (mystery shopper) methodology

A simulated patient, also known as a standardized patient, pseudo patient or mystery shopper, is an individual trained to role play a particular scenario in a particular setting [11]. The mystery shopper methodology is unique as it involves direct observation of events and behaviors in the natural non-controlled setting [11].

In recent years, there has been an increase in asthma incidence in developing countries, possibly due to urbanization [12]. The burden of asthma in the United Arab Emirates (UAE) and the extent to which guidelines to manage asthma are followed is unknown. What is known, however, is that the prevalence of asthma is increasing and that the present level of asthma control in the UAE is suboptimal [12]. Community pharmacies in the UAE offer an accessible source of medications to care for consumers with a variety of healthcare needs. They are either small independently owned or chain franchised shops located on a street or in a shopping mall [13]. In the UAE, medications are classified into one of four categories in relation to their dispensing status: those requiring a prescription to be dispensed, those dispensed only by a pharmacist but without a prescription, over the counter (OTC) medications, and those sold in pharmacy and non-pharmacy outlets. In the UAE, albuterol (salbutamol) in an inhalation form is dispensed by a pharmacist without the need for a prescription, used to relieve symptoms of asthma [14]. While the studies documenting pharmacist role in achieving better outcomes for patients with asthma come from the developed world [5–9], the evidence from developing countries such as the UAE is scarce.

As the evidence about the level of care provided by community pharmacists in the UAE to patients with asthma is currently lacking, the objective of this study was to assess community pharmacists’ skills in assessing, managing, and educating patients with asthma in the community using simulated patient methodology. Findings from this research will help define training needs of community pharmacists in the provision of pharmaceutical care to patients with asthma and will identify key areas needing intervention.

## Methods

### The simulated patients (mystery shoppers)

Three fifth year bachelor of pharmacy female students underwent standardized training to role play a simulated patient and perform the scenario consistently. They watched video recordings of the scenario involving a mock pharmacist and patient, practiced through role play and were given feedback on their performance. All three simulated patients enacted the same scenario. Each pharmacy was visited by one simulated patient, at different times of the day, all days of the week. The simulated patient specifically asked to be seen by a professional pharmacist, not a technician or an assistant.

### Description of the scenario

The scenario (Additional file 1: Appendix 1) described a 20-year-old college student who had asthma for years but in the last 4 months, she had been having frequent awakenings at night because of cough. The cough was described as dry, non-productive and accompanied by wheezing. She had been managing it with different types of cough syrups but they had only marginally helped. She had also been using her albuterol inhaler more frequently, almost three times per day. She was here to request more cough syrup and a new albuterol inhaler, without prescription. The scenario was based on predetermined plots to ensure standardization of pharmacist—patient communication. If asked by the pharmacist, she reported she was being treated with Fluticasone propionate/Salmeterol inhaler, but only used it when the albuterol did not work. If the pharmacist failed to ask questions or provide appropriate information on asthma medications, and the inhaler technique, then, the simulated patient would prompt the pharmacist to provide it by asking the questions: “Can you please tell me the difference between the medications I am using and, “I am not sure if I am using my inhalers correctly, can you please show me how to use them?”.

The asthma in this patient was meant to be classified as *uncontrolled* according to the Global Initiative for Asthma [1]. The correct outcome for this scenario was referral to a physician for appropriate follow up, that the albuterol inhaler was dispensed, while the cough syrup was not. The pharmacist was expected to follow the patient handling scheme described in Additional file 2: Appendix 2.

### Documentation after the visit

After each visit, the “simulated patient” exited the pharmacy and immediately documented details of her encounter with the pharmacist on the Data collection form which contained items relating to patient Assessment, Management following the Herrier approach [10], and on Advice giving by the pharmacist. Specifically, measures of incidence of cough syrup and reliever inhaler supply, physician referral rate, incidence and extent of counseling on the medications, correction of inhaler technique, incidence and extent of counseling on asthma, its triggers, and adherence to the medications and adverse drug reactions were calculated.

The quality of information provided by the pharmacist (whether voluntarily provided or prompted by the simulated patient) was rated in accordance with a coding scheme developed by study investigators based on the Global Initiative for Asthma guidelines [1]. Complete and accurate information covered by the pharmacist = 1,e.g., the pharmacist provided information on shaking the device (if needed), on actuating and breathing coordination, repeat dosing, cleaning the inhaler, remaining doses, and on possible side effects from the medication. Incomplete or only basic information covered by the pharmacist = 2, e.g., the pharmacist only provided information on shaking the device (if needed), and on actuating and breathing coordination. Poor or incorrect information provided by the pharmacist = 3, e.g., the pharmacist provided incorrect information such as recommending the float test to find out when to replace a metered dose inhaler.

### Pilot study

A small pilot study of 10 visits to community pharmacies was conducted. Depending on the feedback from this pilot study, the scenario was modified, items on the Data collection form were adjusted to suit the scenario, and the coding scheme (including interrater coding) for the quality of provided information by the pharmacist was discussed and further refined. Results obtained in the pilot were excluded from data analysis.

### Setting and sampling

Data collection was carried out between March and July 2015. A list of all registered community pharmacies in the emirates of Abu Dhabi. Dubai, Sharjah, and Ajman was compiled from the Yellow Pages directory. The sample size was calculated at 90% confidence level, a 5% margin of error, sample proportion at 0.5 for a population of pharmacies *N* = 1100, to give 214 pharmacies. Randomization was done by importing the list of all pharmacies into Microsoft Excel. The RAND function [15] was used to assign a random number to every pharmacy. As the required sample size equated to approximately one 5th of all pharmacies, to ensure representativeness, one fifth of all pharmacies within each location on the list was visited.

### Data analysis

Data were entered into Microsoft Excel 2016 for Windows (Microsoft Corp, Redmond, WA, USA). Descriptive statistics was carried out using IBM SPSS Statistics 23.0 (SPSS Inc., Chicago, IL, USA). Pearson's chi-squared analyses were performed on cross tabulated data to determine statistically significant (*P* < 0.05) association between any form of assessment (incidence of cough syrup and reliever inhaler supply, physician referral rate, correction of inhaler technique, incidence of counseling on asthma and its triggers, and incidence of counseling on adherence to maintenance therapy and adverse drug reactions), and the pharmacy and pharmacist characteristics: pharmacy type (Independent or Chain), location (Abu Dhabi, Dubai, Sharjah or Ajman) and gender of the pharmacist. Differences between ratings of study investigators who evaluated all visits to the pharmacies were examined using Cohen’s Kappa, a value > 0.7 was considered acceptable to indicate reliability.

## Results

Fourteen pharmacies were not available at the addresses listed, and five pharmacists recognized the visit as part of the study, leading to 195 pharmacies (81 in Abu Dhabi, 50 in Dubai and 64 in Sharjah and Ajman) being visited. Table 1 shows characteristics of pharmacists and pharmacies that were possible to collect.

**Table 1:** Characteristics of pharmacists and pharmacies, *N* = 195

Gender	No (%)
Male	125 (64)
Female	70 (36)
Emirate	No (%)
Abu Dhabi	81 (41)
Dubai	50 (26)
Sharjah	49 (25)
Ajman	15 (8)
Location	No (%)
Shopping center	45 (23)
Street	150 (77)
Pharmacy type	No (%)
Independent	86 (44)
Chain	109 (56)

### First impression and assessment of control

Fifty three (27%) pharmacists asked about the need for cough syrup, and 144 (59%) asked about the need for the albuterol inhaler on the initial request for the medications (Table 2). Most pharmacists 118 (61%) asked if the patient had previously been diagnosed with asthma. Only 51 (26%) asked about other medications the patient was taking for asthma.

**Table 2:** Pharmacist assessment of simulated patient

No	Question of assessment	No (%) *N* = 195
First impression (assessing diagnosis of asthma) & degree of *Control*		
1	Pharmacist asked about cough syrup?	53 (27.2)
2	Pharmacist asked about albuterol inhaler?	144 (58.5)
3	Pharmacist asked about previous diagnosis of asthma?	118 (61)
4	Pharmacist asked about any other medications for asthma?	51 (26)
Assessing *Compliance* with medications		
5	Pharmacist assessed adherence to usual preventer medication use?	38 (19.5)
6	Pharmacist asked about inhaler technique?	1 (0.5)
Assessing *Complications* from disease & medications		
7	Pharmacist assessed disease-related problems, e.g., cough, shortness of breath, chest tightness, etc	53 (27.2)
8	Pharmacist assessed drug-related problems, e.g., hoarseness (Preventer), tachycardia (albuterol)?	32 (16)
Pharmacist *Management* of patient		
9	Pharmacist supplied either one of the medications?	131 (67)
	Both medication	109 (56)
	Only cough syrup	15 (7.7)
	Only albuterol	7 (3.6)
10	Pharmacist referred patient to physician?	126 (65)
Pharmacist *education and advice giving*		
11	Pharmacist provided any information about medications for asthma, what type of information?	56 (29)
	Complete information	1 (0.5)
	Incomplete information	27 (14)
	Poor information	28 (14)
12	Pharmacist provided any counseling on inhaler technique? What type of counseling?	41 (21)
	Complete information	14 (7)
	Incomplete information	25 (13)
	Poor information	2 (1)
13	Pharmacist provided any information about asthma and its trigger factors, what type of information?	16 (8)
	Complete information	4 (2)
	Incomplete information	10 (5)
	Poor information	2 (1)
14	Pharmacist provided information on adherence to preventer medications	1 (0.5)
15	Pharmacist provided information on drug-related problems	32 (16)
Patient *Concerns*: Pharmacist *education and advice giving*		
16	When asked, pharmacist provided information on asthma medications? How well and complete was the counseling?	*N* = 139
	Complete information	42 (30)
	Incomplete information	45 (32)
	Poor information	53 (38)
17	When asked, pharmacist provided comparison of medication use in asthma?	*N* = 195
	Complete information	39 (20)
	Incomplete information	103 (53)
	Poor information	53 (27)
18	When asked, pharmacist counseled on appropriate inhaler technique? How well was the counseling?	*N* = 154
	Complete information	75 (49)
	Incomplete information	53 (34)
	Poor information	26 (17)

### Assessment of compliance

Table 2 also shows that 38 (< 20%) of pharmacists asked if the patient was regularly using a preventer medication and if she was adhering to it, while only *one* pharmacist asked about the inhaler technique the patient uses.

### Assessment of complications

About 53 (27%) asked about severity and frequency of the cough, shortness of breath and chest tightness related to asthma, etc. Only 32 (16%) of the pharmacists asked if the patient was having any side effects from the medications (Table 2).

### Pharmacist management

Table 2 also shows that 109 (56%) supplied both medications, while seven pharmacists appropriately supplied only the albuterol inhaler. The majority 126 (65%) referred the patient to a physician for proper management.

### Advice giving

Only *one* pharmacist gave complete information, while 27 (14%) gave incomplete and another 28 (14%) gave poor information about the medications for asthma. Only 41 (21%) gave information about inhaler technique which was mostly incomplete. Sixteen (8%) pharmacists gave information about asthma and its triggers which was mostly incomplete. Likewise, counseling on adherence to preventer medications was carried out by *one* pharmacist and on side effects by 32 (16%) which was predominantly on hoarseness as a side effect from corticosteroid use.

Table 3 also shows that when pharmacists were prompted to give the information, it was mostly incomplete; < 50% gave complete information about inhaler technique.

**Table 3:** Influence of type of pharmacy on asthma assessment

Question of assessment	Pharmacy Type	Pharmacists addressing question No (%)	Total	*P*-value
Pharmacist asked why need cough syrup?	Indep.	38 (72)	53	0.008
	Chain	15 (28)		
Pharmacist asked why need an albuterol inhaler?	Indep.	76 (53)	144	0.016
	Chain	68 (47)		
Pharmacist asked about possible diagnosis of asthma?	Indep.	75 (63)	118	0.002
	Chain	43 (37)		
Pharmacist asked about any other medications for asthma?	Indep.	36 (71)	51	0.01
	Chain	15 (29)		
Pharmacist assessed adherence to usual preventer medication use?	Indep.	21 (51)	38	0.1
	Chain	17 (46)		
Pharmacist supplied either one of the medications?	Indep.	75 (57)	131	0.2
	Chain	56 (43)		
Pharmacist referred to a doctor?	Indep.	79 (63)	126	0.05
	Chain	47 (37)		
Pharmacist provided any information about medications for asthma	Indep.	44 (79)	56	0.04
	Chain	12 (21)		
Pharmacist provided any counseling on inhaler technique	Indep.	22 (54)	41	0.3
	Chain	19 (46)		
Pharmacist provided any information about asthma and its trigger factors	Indep.	7 (44)	16	0.4
	Chain	9 (56)		

### Pearson's chi-squared analysis

The Pearson's chi-squared test showed that independent pharmacists (Table 3) and pharmacists practicing in Abu Dhabi and Dubai (Table 4) were likely to have a more thorough assessment of the simulated patient. Gender of the pharmacist did not have a significant effect on any variables of assessment, *P* > 0.05. Inter-rater reliability between simulated patients was found at good agreement (Cohen’s Kappa = 0.78).

**Table 4:** Impact of pharmacy location on asthma assessment

Question of assessment	Location	Pharmacists addressing question No (%)	Total	*P*-value
Pharmacist asked why need cough syrup?	Abu Dhabi	23 (43)	53	0.000
	Dubai	18 (34)		
	Sharjah	10 (19)		
	Ajman	2 (4)		
Pharmacist asked why need an albuterol inhaler?	Abu Dhabi	70 (49)	144	0.02
	Dubai	30 (21)		
	Sharjah	30 (21)		
	Ajman	14 (9)		
Pharmacist asked about possible diagnosis of asthma	Abu Dhabi	60 (51)	118	0.04
	Dubai	33 (28)		
	Sharjah	14 (12)		
	Ajman	11 (9)		
Pharmacist asked about any other medications for asthma?	Abu Dhabi	25 (49)	51	0.05
	Dubai	15 (29)		
	Sharjah	10 (20)		
	Ajman	1 (2)		
Pharmacist supplied either one of the medications?	Abu Dhabi	68 (52)	131	0.08
	Dubai	28 (21)		
	Sharjah	30 (23)		
	Ajman	5 (4)		
Pharmacist assessed adherence to usual preventer medication use?	Abu Dhabi	18 (47)	38	0.1
	Dubai	14 (37)		
	Sharjah	5 (13)		
	Ajman	1 (3)		
Pharmacist referred to a doctor?	Abu Dhabi	62 (49)	126	0.2
	Dubai	35 (28)		
	Sharjah	19 (15)		
	Ajman	10 (8)		
Pharmacist provided any information about medications for asthma	Abu Dhabi	22 (40)	56	0.04
	Dubai	22 (39)		
	Sharjah	9 (16)		
	Ajman	3 (5)		
Pharmacist provided any counseling on inhaler technique	Abu Dhabi	19 (46)	41	0.15
	Dubai	15 (37)		
	Sharjah	5 (12)		
	Ajman	2 (5)		
Pharmacist provided any information about asthma and its trigger factors	Abu Dhabi	4 (21)	16	0.2
	Dubai	7 (52)		
	Sharjah	4 (23)		
	Ajman	1 (4)		

## Discussion

In this study, the Herrier approach utilized to assess community pharmacist handling of asthmatic patients has offered a comprehensive platform to assess pharmacists’ competence and define shortcomings and hence training needs of pharmacists in managing patients with asthma.

It was clear that assessment of *Control* of asthma by community pharmacists was suboptimal in this study; many pharmacists did not really question the need or use of the cough syrup or the albuterol inhaler, many others did not ask about a diagnosis for asthma or the medications that truly treat it. Pharmacists are ideally positioned to assess control of asthma and to contribute to improving it using simple efficient measures, such as questioning the patient or administering an Asthma Control Test [16]. Community pharmacies are also ideally positioned as a suitable venue for screening patients for a possible diagnosis of asthma [17]. Pharmacists in this study would have missed a possible diagnosis of asthma had the scenario described a patient with no prior diagnosis. Training pharmacists in screening and assessment of asthma control is clearly indicated.

On the assessment of *Compliance*, only *one* pharmacist in this study asked if the patient was adhering to her preventer medication. Additionally, a small number of pharmacists asked if the patient was using her inhaler correctly, this was where pharmacists could positively impact patient use of their medications, considering that up to 57% of patients with asthma do not use their inhalers properly [18]. Basheti et al. [19] had established clinical and quality of life improvements when pharmacists provided regular counseling on inhaler technique. Pharmacists’ involvement increased adherence to regular inhaled corticosteroid medications and improved asthma control in poorly controlled non-adherent patients [11]. Pharmacists in the UAE do not counsel on the use of inhalers and devices [20], many of them cited lack of patient demand for the services as a reason for not providing them [13]. However, evidence suggests patients have high expectations of and willingness to use community pharmacy services such as counseling on asthma devices and inhalers if provided [21]. The findings suggest pharmacists should provide services related to asthma considering its increased prevalence, suboptimal level of control and patient willingness to receive the services.

On the assessment of *Complications*, only 32 pharmacists asked about side effects from medications, patients using cough syrup may be exposed to side effects such as headaches, nausea and vomiting, dry secretions, dizziness and sleepiness, and possibly anticholinergic effects depending on the type of antitussive medication [22]. Frequent albuterol use is associated with side effects such as tachycardia, palpitations, tremor, headaches and more seriousones such as cardiomyopathies [23]. Oropharyngeal disorders such as hoarseness, tingling and mouth irritation represent the most frequent side effect of inhaled steroids whose occurrence is positively associated with decreased adherence to the medications [24]. It is important that pharmacists are trained to assess the occurrence of these side effects and offer patients solutions to deal with them.

On the assessment of *Management*, the majority of pharmacists (56%) supplied both of the cough syrup and albuterol on patient demand. This implied that the majority of the pharmacists might have not recognized that the cough was related to the uncontrolled asthma. It was also possible that the pharmacists thought the cough was part of a common cold/flu episode for which they normally dispensed cough syrup without much questioning. Most pharmacists correctly supplied albuterol inhaler to the patient as they might have felt the patient needed it until she had seen a physician. Unfortunately, patients might not actually go to see their physician and continue to shop for medications that will only help with symptoms; which could put them at a high risk of major complications and poor quality of life. This is especially relevant, where patients do not have access to adequate primary care services and depend heavily on pharmacy services for their medical needs [20].

On the assessment of *Advice giving*, even when prompted, a small percentage of pharmacists adequately counselled the patient on the medications and the inhaler technique, responsibilities conventionally expected of pharmacists. These findings are commonly reported in the literature, in a study from Australia measuring pharmacist assessment and counseling on the supply of albuterol inhaler in community pharmacies [25], only 24% counselled on medications, while no pharmacist counselled on inhaler technique. Studies from the region mirror this, a study from Sudan found major deficiencies in pharmacist ability to demonstrate proper inhaler technique [26]. Although unfounded [21, 27], many pharmacists are paternalistic and avoid giving information to patients. This highlights the importance of improving patient education and proactivity in demanding the information from pharmacists as the pharmacists may elect not to give it [28]. Increased negative patient outcomes from lack of education far more justifies the need to give advice.

Our study also found that community pharmacists working in independent pharmacies were likely to assess the asthmatic patient more thoroughly. Similar findings were reported in a previous study from the UAE; pharmacists practicing in independent pharmacies were more likely to be involved in providing enhanced professional services compared to those practicing in chain pharmacies [20]. Rather than engaging in enhanced professional services, it is possible that pharmacists practicing in chain pharmacies were under more pressures to meet profit margins and sales quotas expected from them by corporate management [13]. Unfortunately, this practice prevails in the UAE and other countries in the region leading to a “business” image to the profession that might be contributing to the low patient demand for services by pharmacists as reported earlier [13].

Findings suggest the compelling need for improved pharmacist handling of patients with asthma. Empirically, adequate asthma training should be incorporated into pharmacy curricula so new pharmacists graduate with needed skills [29]. Post graduate pharmacist training that depends on hands on experiences, involving patient simulations of scenarios encountered in practice with constructive feedback has been shown successful [3]. Multidisciplinary credentials through asthma education certification have been shown to increase the credibility of pharmacist provided asthma education and encourage development of pharmacist skills in the management of asthma [30]. Preferably, interventions to train pharmacists are directed to provide services that are simple to implement, and have the flexibility for pharmacists to adapt the services to meet local requirements [31], as defined in this study. It is essential that those involved in training of pharmacists at the pre-registration level and those involved in designing continuing pharmacy education consider the findings in this study in the design of pharmacy courses and continuing education programming to assure pharmacists obtain the necessary competence in managing asthma.

Pharmacists have previously cited reasons for not providing enhanced services in community pharmacies in the UAE to include shortage of staff and lack of time to deliver the services, but equally important is their inability to charge for the services [13, 20]. Drug policies and regulation currently in effect in the Country prohibit pharmacists from billing patients for non-product services including screening for disease, health promotion interventions and counseling on medications [13]. It is imperative that these drug policies are reviewed again to allow pharmacists to charge for cognitive services as this will encourage them to offer care to their patients the same way they offer products.

## Limitations

Much effort was exerted to train the simulated patients and reduce their own individual influence on the data collected. Use of students as simulated patients may be a limitation; however, extensive training of the students was used to enhance their skills. Pharmacists in independent pharmacies asked more questions and provided better assessment of the simulated patient in this study, it was not known if this effect was confounded by factors such as the age of the pharmacists who may be older and more experienced than those working in chain pharmacies. Due to the simulated patient—nature of the study, it was not possible to obtain information regarding the age or experience of the pharmacists which would have allowed for the exploration of their effect on the findings.

## Conclusion

This study has revealed several shortcomings in the ability of pharmacists to assess control, management and advice giving related to asthma, and the medications and devices that treat it. This research has informed pharmacist education and training needs in several aspects of asthma handling, and has highlighted the importance of improving patient education and demand for services related to their condition. The study has also informed system and policy interventions that will help in improving quality of care provided by the community pharmacists to asthmatic patients.

## Supplementary Information

**Additional file 1: Appendix 1.** Patient scenario.

**Additional file 2: Appendix 2.** Patient handling scheme.

## Data Availability

Please contact author for data requests.

## Abbreviations

OTC: Over the counter
SPSS: Statistical package for the social sciences
UAE: United Arab Emirates
USA: United States of America

## References

[1] Global Initiative for Asthma (GINA). GINA report, global strategy for asthma management and prevention. www.ginasthma.org. Accessed 5 Oct 2018.

[2] The global asthma network. The global asthma report 2018. Available at http://www.globalasthmareport.org/Global%20Asthma%20Report%202018.pdf. Accessed 8 June 2019.

[3] Saini B, Smith P, Armour C, Krass I (2006). An educational intervention to train community pharmacists in providing specialized asthma care. Am J Pharm Educ.

[4] Office of disease prevention and health promotion. Healthy people 2020. Available at: http://www.healthypeople.gov/2020/topicsobjectives2020/objectiveslist.aspx?topicId=36 Accessed 12 Mar 2018.

[5] Cordina M, McElnay JC, Hughes CM (2001). Assessment of a community pharmacy based program for patients with asthma. Pharmacotherapy.

[6] McLean W, Gillis J, Waller R (2003). The BC Community Pharmacy Asthma Study: a study of clinical, economic and holistic outcomes influenced by an asthma care protocol provided by specially trained community pharmacists in British Columbia. Can Respir J.

[7] Narhi U, Airaksinen M, Tanskanen P (2000). Therapeutic outcomes monitoring by community pharmacists for improving clinical outcomes in asthma. J Clin Pharm Ther.

[8] Barbanel D, Eldridge S, Griffiths C (2003). Can a self-management programme delivered by a community pharmacist improve asthma control? A randomized trial. Thorax.

[9] Emmerton L, Shaw J, Kheir N (2003). Asthma management by New Zealand pharmacists: a pharmaceutical care demonstration project. J Clin Pharm Ther.

[10] Herrier R, Apgar D, Boyce R, Foster S. Obtaining a patient history. In: Patient assessment in pharmacy. Chapter 2. New York: McGraw-Hill Education; 2015.

[11] Berger K, Eickhoff C, Schulz M (2005). Counselling quality in community pharmacies: implementation of the pseudo customer methodology in Germany. J Clin PharmTher.

[12] Tarraf H, Aydin O, Mungan D (2018). Prevalence of asthma among the adult general population of five Middle Eastern countries: results of the SNAPSHOT program. BMC Pulm Med.

[13] Hasan S, Suleiman H, Chapman CB, Stewart K, Kong DCM (2011). Community pharmacy in the United Arab Emirates: characteristics and workforce issues. Int J Pharm Pract.

[14] Reclassification and revised dispensing mode. HAAD. 2011. www.haad.ae/HAAD/LinkClick.aspx?fileticket=8YIa2Sz8nDE%3D&tabid=207. Accessed 26 Apr 2019.

[15] The Excel Rand Function. Exceljet. 2019. www.exceljet.net/excel-functions/excel-rand-function. Accessed 26 Apr 2019.

[16] Guénette L, Breton MC, Grégoire JP, Bolduc SY, Boulet LP, Dorval E, Moisan J (2015). Effectiveness of an asthma integrated care program on asthma control and adherence to inhaled corticosteroids. J Asthma.

[17] Elliott J, Harrison C, Konopka C, Wood J, Marcotullio N, Lunney P, Skoner D, Gentile D (2015). Pharmacist-led screening program for an inner-city pediatric population. JAPHA.

[18] Sanchis J, Gich I, Pedersen S (2016). Systematic review of errors in inhaler use: has patient technique improved over time?. Chest.

[19] Basheti I, Reddel H, Armour C, Bosnic-Anticevich S (2007). Improved asthma outcomes with a simple inhaler technique intervention by community pharmacists. J Allergy Clin Immunol.

[20] Hasan S, Suleiman H, Stewart K, Kong DCM, Chapman CB (2012). Community pharmacy services in the UAE. Int J Pharm Pract.

[21] Hasan S, Suleiman H, Stewart K, Kong DCM, Chapman CB (2015). Patient expectations of and willingness to use primary care pharmacy services in the United Arab Emirates. Int J Pharm Pract.

[22] Smith SM, Schroeder K, Fahey T (2014). Over-the-counter (OTC) medications for acute cough in children and adults in community settings. Cochrane Database Syst Rev.

[23] Abramson MJ, Walters J, Walters EH (2003). Adverse effects of beta-agonists: are they clinically relevant?. Am J Respir Med.

[24] Molimard M, Le Gros V, Robinson P, Bourdeix I (2010). Prevalence and associated factors of oropharyngeal side effects in users of inhaled corticosteroids in a real-life setting. J Aerosol Med Pulm Drug Deliv.

[25] Schneider CR, Everett AW, Geelhoed E, Kendall PA, Clifford RM (2009). Measuring the assessment and counseling provided with the supply of nonprescription asthma reliever medication: a simulated patient study. Ann Pharmacother.

[26] Osman A, Hassan ISA, Ibrahim MI (2012). Are Sudanese community pharmacists capable to prescribe and demonstrate asthma inhaler devices to patrons? A mystery patient study. Pharm Pract.

[27] Hasan S, Suleiman H, Stewart K, Kong DCM, Chapman CB, Hasan MY (2013). Assessing patient satisfaction with community pharmacy in the UAE using a newly-validated tool. Res Soc Admin Pharm.

[28] Peters J, Desai K, Ricci D, Chen D, Singh M, Chewning B (2016). The power of the patient question: a secret shopper study. Patient Educ Counsel.

[29] Hasan S, Al Sabbagh R, AlHumaidi R, AlMallah M, Khan F (2016). Objective Structured Clinical Examination (OSCE) in assessing pharmacy students’ Arabic language competence in asthma management. Pharm Educ.

[30] National Community Pharmacist Association. Independent pharmacies take top honors in consumer reports nationwide survey. 2003. www.ncpanet.org/. Accessed 21 Oct 2018.

[31] Thornley T, Gray N, Anderson C, Eastham S (2006). A study to investigate the extent of delivery of an intervention in asthma, in a UK national community pharmacy chain, using mystery customers. Patient Educ Counsel.

