# Supplementary material for: A simulated patient study to evaluate community pharmacist assessment, management and advice giving to patients with asthma
Source: J Pharm Policy Pract. 2021 Jan 12;14:8. doi: 10.1186/s40545-020-00294-4 (PMC7805111; doi:10.1186/s40545-020-00294-4)
Supplement: Supplementary file 1 — Additional file 1: Appendix 1. Patient scenario. [file 40545_2020_294_MOESM1_ESM.docx]

**Appendix 1**

**Patient Scenario**

You are a 20 y.o. college student who has had asthma for years but in the last 4 months you have been having frequent awakenings at night because of cough. You have been managing it with different types of cough syrups but they have marginally helped. You are requesting a cough syrup and would like to know how much and how often you need to use it. You also have been using your albuterol inhaler more frequently now, almost three times per day, and you’re here to buy a new one. Upon questioning, you would also be treated with fluticasone/salmeterol (Seretide) inhaler but you use that once in a while when your albuterol does not work. Upon questioning, you have allergies but you’re not sure how they contribute to your asthma. You are also uncertain about the use of your inhaler, upon questioning, you forget to shake the device before use and your inhalation technique is completely wrong.

If the pharmacist did not ask about why you’re purchasing the cough syrup and the albuterol inhaler, you are wondering why you are needing to use these medications so frequently now.

If the pharmacist did not ask about the inhaler technique, ask if you are using it appropriately!

If the pharmacist does not ask about other medications or appropriateness of medications, you are wondering if you are using this other medication “Seretide” appropriately and at the right time!

If the pharmacist does not cover any counseling on medications, ask what the difference between the two inhalers!

1. Did the pharmacist ask about why you need cough syrup?
2. Did the pharmacist ask about why you need an albuterol inhaler?
3. Did the pharmacist supply either one of the medications?
4. Did the pharmacist ask about possible diagnosis of asthma?
5. Did the pharmacist ask that you refer to a doctor?
6. Did the pharmacist ask about any other medications for asthma?
7. Did the pharmacist ask about your inhaler technique?
8. Did the pharmacist provide any counseling on inhaler technique? What type of counseling?
9. Did the pharmacist provide any information about asthma and its trigger factors, what type of information?
10. Did the pharmacist provide any information about medications for asthma, what type of information?
11. When asked “I am not sure if I am using my inhalers correctly, can you please show me how to use them?” Did the pharmacist counsel on appropriate inhaler technique? How well was the counseling?
12. When asked, “Can you please tell me the difference between the medications I am using?” Did the pharmacist provide medication information on asthma? How well and complete was the counseling?
13. When asked, did the pharmacist provide comparison of medication use in asthma?
14. Did the pharmacist assess or provide counseling on adherence to usual preventer medication use?
15. Did the pharmacist assess or provide counseling on drug-related problems such as hoarseness of voice, thrush, from Seretide, tachycardia from albuterol?
