# Supplementary material for: A simulated patient study to evaluate community pharmacist assessment, management and advice giving to patients with asthma
Source: J Pharm Policy Pract. 2021 Jan 12;14:8. doi: 10.1186/s40545-020-00294-4 (PMC7805111; doi:10.1186/s40545-020-00294-4)
Supplement: Supplementary file 2 — Additional file 2: Appendix 2. Patient handling scheme. [file 40545_2020_294_MOESM2_ESM.docx]

**Appendix 2**

**Patient handling scheme**

1. ***Pharmacist Assessment of the simulated patient covered questions:***

*First impression*

- If the pharmacist asked about why the need for cough syrup and/or the albuterol inhaler; this assesses pharmacists’ screening for a possible diagnosis of asthma, and the degree of asthma control in the diagnosed patient.
- If the pharmacist asked about disease history and previous diagnosis of asthma, this assesses pharmacists’ confirmation of asthma.

*Pharmacist Assessment of the* *3 C’s:*

*Control*:

1. If the pharmacist asked about the degree of control of symptoms through assessing the need of cough syrup
2. If the pharmacist asked about frequency of reliever medication use (as a marker of control of asthma)
3. If the pharmacist asked about any other medications for asthma that the simulated patient is using (preventer medications, in this case fluticasone/salmeterol inhaler)

*Compliance:*

1. If the pharmacist assessed adherence to usual preventer medication use

2) If the pharmacist asked about the simulated patient’s inhaler technique

*Complications*

1. If the pharmacist asked about complications of the disease (carried out also in *Control* assessment above)
2. If the pharmacist assessed drug-related problems from asthma medications such as any side effects from frequent albuterol use or preventer medications (fluticasone/salmeterol)
3. ***Pharmacist Management of the simulated patient covered questions:***
4. If the pharmacist supplied either of the cough syrup and/or the albuterol inhaler upon the request of the simulated patient.
5. If the pharmacist asked about any other medications for asthma that the simulated patient is using as mainstay treatment for asthma (carried out also in *Control* assessment above).
6. If the pharmacist referred to a physician for proper management of the asthma.
7. ***Pharmacist education and advice giving covered questions:***
8. If the pharmacist provided any information about medications for asthma including comparison of the medication.
9. If the pharmacist provided any counseling on inhaler technique
10. If the pharmacist provided any information about asthma and its trigger factors
11. If the pharmacist provided counseling on adherence to usual preventer medication use
12. If the pharmacist provided counseling on drug-related problems

It was selected that the pharmacist would also address the 4^th^ C: *Concerns* of the patient, so if the pharmacist failed to ask if the patient had any concerns about their condition and/or its treatment, or provide advice on their medications, and the proper technique of inhaler use, they would be prompted by the simulated patient who was *concerned* about their medications and the inhaler technique.
